# Association between trajectories of sleep quality and postpartum depression: a group-based trajectory model and computer-simulated network analysis

**DOI:** 10.1186/s12916-026-04689-z

**Published:** 2026-02-10

**Authors:** Xiaoxiao Mei, Jinzhou Yu, Qianru Liu, Yan Li, Shuhan Li, Qianwen Chen, Hongman Li, Ying Xiong, Ranran Mei, Zengjie Ye

**Affiliations:** 1 School of Nursing, Guangzhou Medical University, Guangzhou, China; 2 School of Nursing, The Hong Kong Polytechnic University, Hong Kong, China; 3 School of Nursing, The University of Hong Kong, Hong Kong, China; 4 Women’s Hospital, School of Medicine, Zhejiang University, Hangzhou, China; 5 School of Nursing, Guangzhou University of Chinese Medicine, Guangzhou, China; 6 Department of Breast Oncology, Guangzhou Institute of Cancer Research, The Affiliated Cancer Hospital, Guangzhou Medical University, Guangzhou, China

**Keywords:** Group-based trajectory model, Network analysis, Postpartum depression, Sleep quality

## Abstract

**Background:**

Sleep quality during pregnancy and the postpartum period is increasingly acknowledged as a critical influencing factor of postpartum. However, the complexities of this relationship, particularly the core depressive symptoms across varying sleep quality trajectories, remain poorly understood.

**Methods:**

This study included 372 participants from the “Be Resilient to Postpartum Depression” cohort, with data collected at four intervals spanning early pregnancy to 42 days after childbirth. Validated instruments were used to evaluate both sleep quality and postpartum depression. Data analysis employed group-based trajectory modeling and computer-simulated network analysis.

**Results:**

Two distinct trajectories of sleep quality were identified: “increasingly poor” trajectory (41.4%), which exhibited a markedly higher rate of postpartum depression (*OR* = 2.75, *P* < 0.001), and “stably good” trajectory (58.6%). Within the “increasingly poor” trajectory, the symptom “Things have been getting on top of me” emerged as both the core and aggravating symptom. In the “stably good” group, the core and aggravating symptom was “I have felt scared or panicky for no very good reason.” Additionally, “I have been anxious or worried for no good reason” and “I have been so unhappy that I have had difficulty sleeping” were identified as key symptoms associated with alleviating depressive symptoms in the “increasingly poor” and the “stably good” groups, respectively.

**Conclusions:**

The study underscores the heterogeneous nature of sleep quality trajectories and their distinct associations with postpartum depressive symptoms, highlighting the necessity for tailored mental health interventions.

**Supplementary Information:**

The online version contains supplementary material available at 10.1186/s12916-026-04689-z.

## Background

Postpartum depression (PPD) represents an important public health problem, with a global prevalence estimated at approximately 17.2% [1]. In China, this figure is even higher, at 20.2% [2]. PPD can result in adverse outcomes for both mothers and infants [3, 4], emphasizing the importance of identifying protective factors against PPD. Recent research has focused increasingly on the factors influencing PPD, with sleep quality emerging as a critical element [5].

Sleep quality undergoes considerable fluctuations throughout the pregnancy and postpartum phase [6, 7]. A recent systematic review highlighted substantial heterogeneity in sleep quality, with 12 studies identifying 2 to 4 distinct trajectories [8]. Notably, 11 of these studies concentrated on either the interval covering the third trimester to postpartum or solely on the pregnancy phase [8]. While one study examined sleep quality trajectories from early pregnancy through 42 days postpartum, it was limited by a small sample size of only 101 participants [9]. These aspects underscore the need for further investigation with larger sample sizes to elucidate these dynamics more comprehensively.

Previous research has investigated the link between sleep quality trajectories and PPD. For instance, research has shown that women in the “increasingly poor” and “stably poor” groups are at greater risk for elevated postpartum depressive symptoms compared to those in the “stably good” group, with odds ratios of 12.42 and 21.04, respectively [10]. Another study reported that individuals within the clinical insomnia trajectory exhibited elevated levels of PPD than those in the subclinical or no insomnia trajectory (all *P* < 0.05) [9]. However, these studies mainly employed logistic regression or one-way ANOVA to assess differences in PPD risk across identified sleep quality trajectories, without further exploring how depression manifests in different sleep patterns [9, 10].

Traditionally, research has relied on composite scores from standardized depression measurement questionnaires. While these scores facilitate the identification of overall depression severity, they may mask the complex interplay and variability among individual symptoms [11]. This underscores the importance of adopting innovative analytical methods that can capture the specific PPD symptoms across different sleep quality trajectories. Network analysis provides a promising approach, enabling the visualization of symptom interconnections and the identification of central symptoms within distinct subgroups [12, 13]. However, this approach has not been widely used within the context of PPD relative to varying sleep patterns.

Additionally, although existing literature indicates that specific depressive symptoms may significantly influence overall depression scores [14], their functional dynamics within the postpartum context remain unexplored. The use of computational simulations to evaluate the impacts of specific symptoms on overall depression scores constitutes an innovative advancement [15]. This approach can aid in identifying critical targets for intervention, thereby facilitating more personalized and impactful mental health strategies for postpartum women [15, 16].

Overall, existing studies have established a connection between sleep quality trajectories and PPD, yet notable gaps remain. The current study seeks to fill these gaps by (1) applying a group-based trajectory model to identify potential sleep quality patterns from the first trimester to 42 days postpartum, (2) utilizing network analysis to pinpoint distinct core symptoms across different sleep quality trajectories, and (3) applying computational simulations to target specific postpartum depressive symptoms for intervention.

## Methods

### Study design and participants

This prospective study was conducted within the Be Resilient to Postpartum Depression program (registration number: ChiCTR2100048465), with detailed study design information available in prior reports [17–19]. Four assessments were conducted following relevant clinical guidelines [20–23]: T0 (the first trimester), T1 (second trimester), T2 (third trimester), and T3 (postpartum period). Eligibility requirements included the following: (1) aged 18 years or older, (2) confirmation of pregnancy by ultrasound, and (3) proficiency in Mandarin for communication. Exclusion criteria comprised the following: (1) a diagnosed mental disorder and (2) termination of pregnancy.

**Fig. 1:**
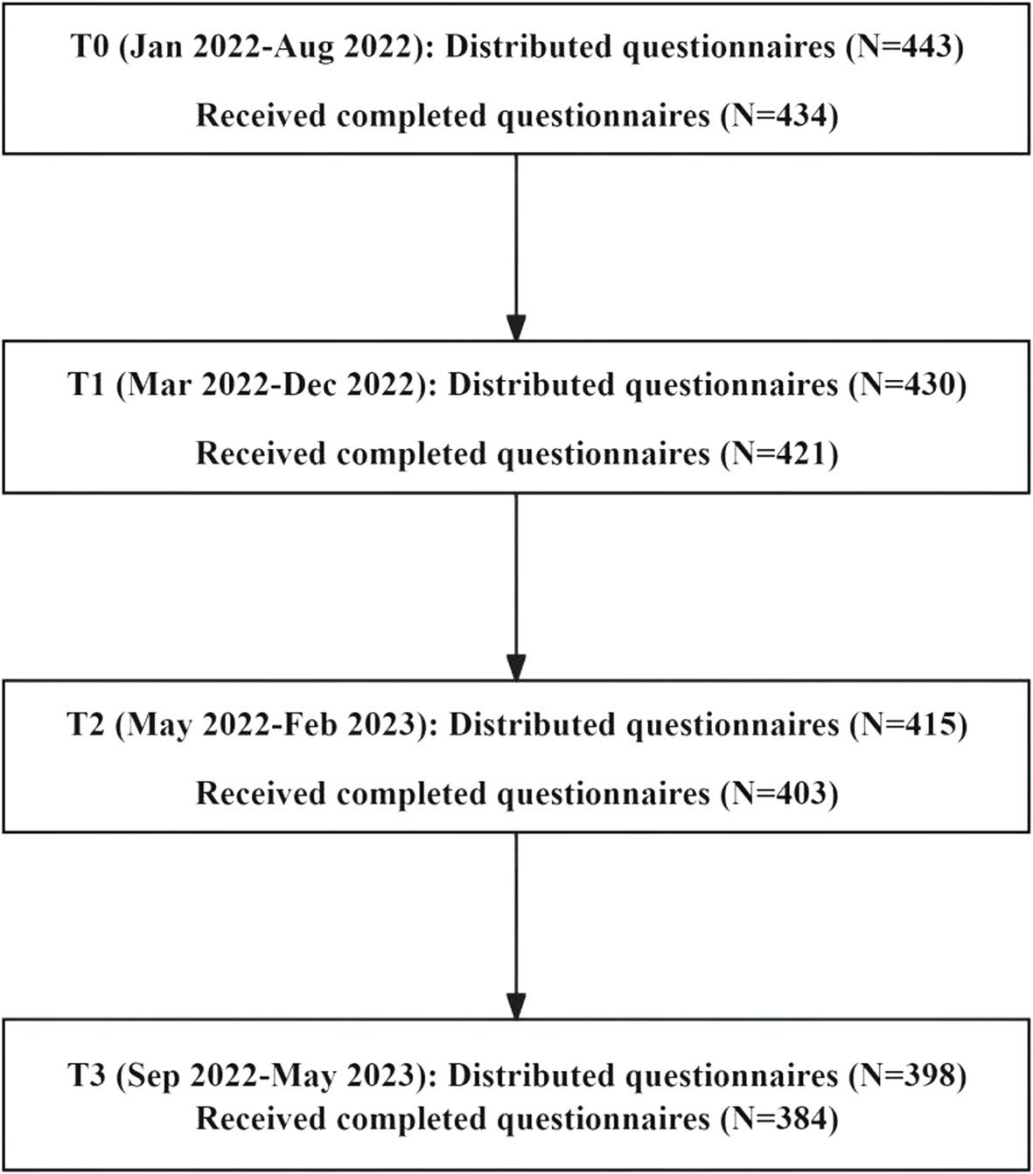
Data collection flowchart

Self-administered questionnaires were completed by participants at the hospital, and researchers were on hand to answer questions if needed. Those lost to follow-up were not included in the final analysis. At T0, 434 of 443 distributed questionnaires were returned (98.0%). At T1, 421 of 430 issued questionnaires were received (97.9%). At T2, 403 of 415 distributed questionnaires were returned (97.1%). Finally, at T3, 384 of 398 provided questionnaires were returned (96.5%). Ultimately, 372 participants completed data across all four time points (see Fig. 1).

According to Nagin (2005) and Jones and Nagin (2007) [24, 25], a total sample size of approximately 300–500 is generally adequate for obtaining stable and reliable estimates when modeling 2 to 4 trajectory groups. Therefore, our sample size of 372 participants falls within this recommended range.

### Measurements

#### Demographic characteristics and pregnancy-related variables

According to previous research [26, 27], we collected the following demographic characteristics: age, level of education, work status, birthplace, marital status, monthly household income, and pregnancy-related variables: nulliparity (yes or no), pregnancy intention (planned or unplanned), and mode of delivery (vaginal or cesarean).

#### Sleep quality

The Pittsburgh Sleep Quality Index (PSQI) was used to assess sleep quality at T0, T1, T2, and T3 [28]. This self-reported tool assesses seven components, yielding an overall score ranging from 0 to 21 [28]. The Chinese adaptation has shown adequate reliability and has been utilized in our prior research [17, 29]. The Cronbach’s alpha values in this study ranged from 0.64 to 0.71.

#### Postpartum depression

Postpartum depression at T3 was evaluated using the Edinburgh Postnatal Depression Scale [30]. This scale includes 10 items scored on a 4-point Likert scale, producing total scores ranging from 0 to 30. A score of 13 or above suggests significant depressive symptoms [31]. The Chinese adaptation has exhibited strong reliability and has been used in our previous study [32, 33]. In this study, the Cronbach’s alpha was 0.90.

### Statistical analysis

First, the distribution of categorical variables was presented as frequencies and percentages, while means and standard deviations (SD) were used for continuous variables.

Next, distinct sleep quality trajectories were distinguished through group-based trajectory modeling (GBTM) [34], a restrictive form that fixes within-class variance to zero and assumes homogeneity within each latent class. This approach focuses on identifying distinct subpopulations that share similar developmental trajectories rather than modeling within-class variability in growth parameters. We fitted models with two to four trajectory groups, sequentially assessing the significance of the intercept, linear, quadratic, and cubic terms of time. The optimal model was selected based on lower Bayesian information criterion (BIC), Akaike information criterion (AIC), log-likelihood (LL), higher entropy, and a minimum group size of 5% per group [34]. Model adequacy was assessed according to three criteria: (1) an average posterior probability of group membership exceeding 0.7, (2) less than 5% the difference between the estimated group probabilities and the observed proportion of group assignments determined by the maximum probability rule, and (3) the odds of correct classification of 5 or greater for all groups [35]. To further validate the accuracy of the trajectory, a sensitivity analysis was conducted that included pregnant women who had at least completed the baseline measurements (*N* = 434). The full information maximum likelihood estimation method, which incorporates all available data without listwise deletion, was used to address missing data.

Following this, differences in EPDS scores between the identified two trajectories were evaluated using a Bayesian independent samples *t*-test, while logistic regression was utilized to explore the odds ratios (OR) of identified trajectories for postpartum depression. As EPDS contains an item (item 7) that assesses sleep difficulty caused by emotion, which may inflate the association between the identified sleep trajectories and depressive symptoms, we conducted an additional linear regression using the total EPDS score with and without item 7 to validate the results. The differences in regression coefficients were assessed using a *z*-test [36].

Finally, high centrality indices calculated by Ising network analysis were used to explore the core symptoms for both groups at 42 days postpartum [37]. Network stability was evaluated using the centrality stability coefficient. A coefficient greater than 0.25 indicates that the stability is within the acceptable range, while a coefficient exceeding 0.5 signifies good stability [38]. The NodeIdentifyR algorithm was applied in a computer-simulated analysis to identify effective intervention targets. In the Ising model, EPDS items were coded as 0 for absence (score of 0) and 1 for presence (scores between 1 and 3) [39]. This algorithm simulates data changes to evaluate the impact of symptom-specific interventions [16].

Statistical analyses were performed using Stata MP 18.0, JASP 0.18.3.0, and R 4.5.0, with significance defined as a two-tailed *P*-value below 0.05.

## Results

### Sample characteristics

The average age of the 372 participants was 29.7 years (*SD* = 4.10), with an average EPDS score of 9.11 (*SD* = 4.38). Among the participants, 70.0% had at least an associate degree, and 72.9% were employed. Additionally, 59.7% were born in rural areas, and half were nulliparous. Most pregnancies (89.3%) were planned, and 61.2% of the deliveries were vaginal. Further details are provided in Table 1.

**Table 1:** Baseline characteristics of participants

	N (%)/mean ± SD	EPDS, mean ± SD
Age, year	29.7 ± 4.10	9.11 ± 4.38
Level of education		
Secondary or below	112 (30.1)	9.38 ± 4.82
Associate degree	139 (37.4)	9.27 ± 4.31
Bachelor or high	121 (32.5)	8.69 ± 4.03
Work status		
Employed	271 (72.9)	9.01 ± 4.10
Unemployed	101 (27.2)	9.38 ± 5.09
Marital status		
Married	351 (94.4)	9.26 ± 4.40
Unmarried	21 (5.7)	6.67 ± 3.38
Monthly household income, CNY		
≤ 4000	92 (24.7)	9.22 ± 4.63
> 4000	280 (75.3)	9.08 ± 4.31
Birthplace		
Urban	150 (40.3)	9.07 ± 3.93
Rural	222 (59.7)	9.14 ± 4.68
Nulliparity		
Yes	186 (50.0)	8.96 ± 4.18
No	186 (50.0)	9.27 ± 4.59
Pregnancy intent		
Planned	332 (89.3)	9.11 ± 4.42
Unplanned	40 (10.8)	9.18 ± 4.11
Delivery mode		
Vaginal	227 (61.2)	8.89 ± 4.46
Cesarean	145 (39.0)	9.46 ± 4.26
Identified trajectories		
Stably good	218 (58.6)	7.88 ± 4.17
Increasingly poor	154 (41.4)	10.86 ± 4.08

### Model selection and sleep quality trajectories

**Fig. 2:**
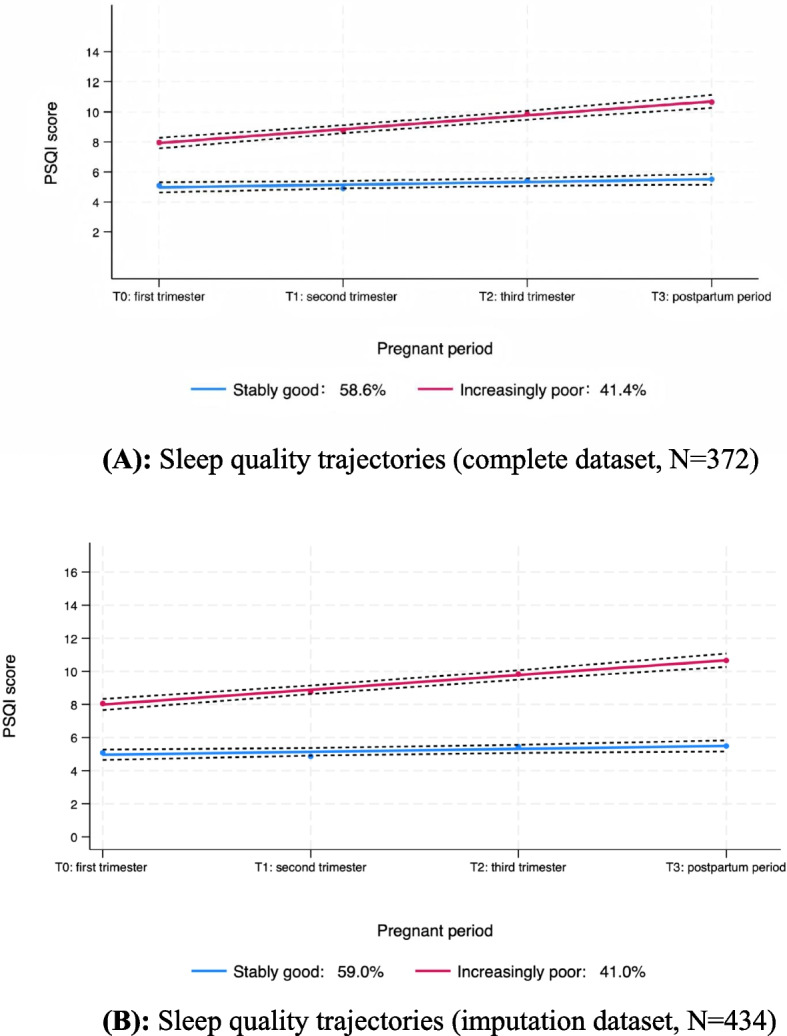
Sleep quality trajectories identified by group-based trajectory modeling. **A** Sleep quality trajectories (complete dataset, *N* = 372). **B** Sleep quality trajectories (imputation dataset, *N* = 434). Note: The scores for the increasingly poor group consistently exceeded 5, suggesting that PSQI scores above 5 are indicative of poor sleepers

The four-group model (polynomial order: 1113) had the lowest absolute BIC value (closest to zero), but its entropy was the lowest compared with the two-group (0.803) and three-group (0.856) models. Neither the four-group nor the three-group model met the minimum group size criterion of 5% per trajectory. Thus, the two-group model (polynomial order: 11) was selected for further analysis, supported by an average group posterior probability of 0.92 or higher for each group, a close match between the estimated group probabilities and the actual group assignments, and odds of correct classification of 10.93 or higher. Details about model comparison and the accuracy of the selected model are presented in Tables 2 and 3, respectively. Figure 2A illustrates two distinct trajectories: “stably good” (58.6%), representing participants with consistently good sleep quality, and “increasingly poor” (41.4%), indicating those whose sleep quality declined from a moderate baseline. Figure 2B shows that the sensitivity analysis using pregnant women who had at least completed the baseline measurements was consistent with the primary trajectory results.

**Table 2:** Model selection results

Number of trajectories	2	3	4
Polynomial order^a^	11	113	1113
BIC (*N* = 1488)^b^	− 3596.65	− 3556.79	− 3542.58
BIC (*N* = 372)^c^	− 3592.49	− 3549.17	− 3532.87
AIC^d^	− 3580.73	− 3527.61	− 3505.44
LL^e^	− 3574.73	− 3516.61	− 3491.44
Entropy	0.803	0.856	0.750
Trajectory proportion (%)	Trajectory 1: 58.60	Trajectory 1: 53.23	Trajectory 1: 39.25
	Trajectory 2: 41.40	Trajectory 2: 41.94	Trajectory 2: 33.06
		Trajectory 3: 4.84	Trajectory 3: 23.39
			Trajectory 4: 4.30

^a^Trajectory shapes: 1, linear; 3, cubic. ^b^*BIC* Bayesian information criterion (for the total number of observations). ^c^*BIC* Bayesian information criterion (for the total number of participants). ^d^*AIC* Akaike information criterion. ^e^*LL* log-likelihood

**Table 3:** Model diagnostics^a^

Trajectories	AvePP^b^	OCC^c^	|EP-P|^d^
Stably good	0.96	23.54	0.01
Increasingly poor	0.92	10.93	0.01

^a^Model polynomial order: 11. ^b^Average group posterior probability. An AvePP greater than 0.7 for all groups is recommended. ^c^Odds of correct classification. An OCC of five or more is recommended for all groups. ^d^*EP* estimated group probabilities; *P* proportion assigned to the group using the maximum probability rule

### The association of sleep quality trajectories with postpartum depression

**Fig. 3:**
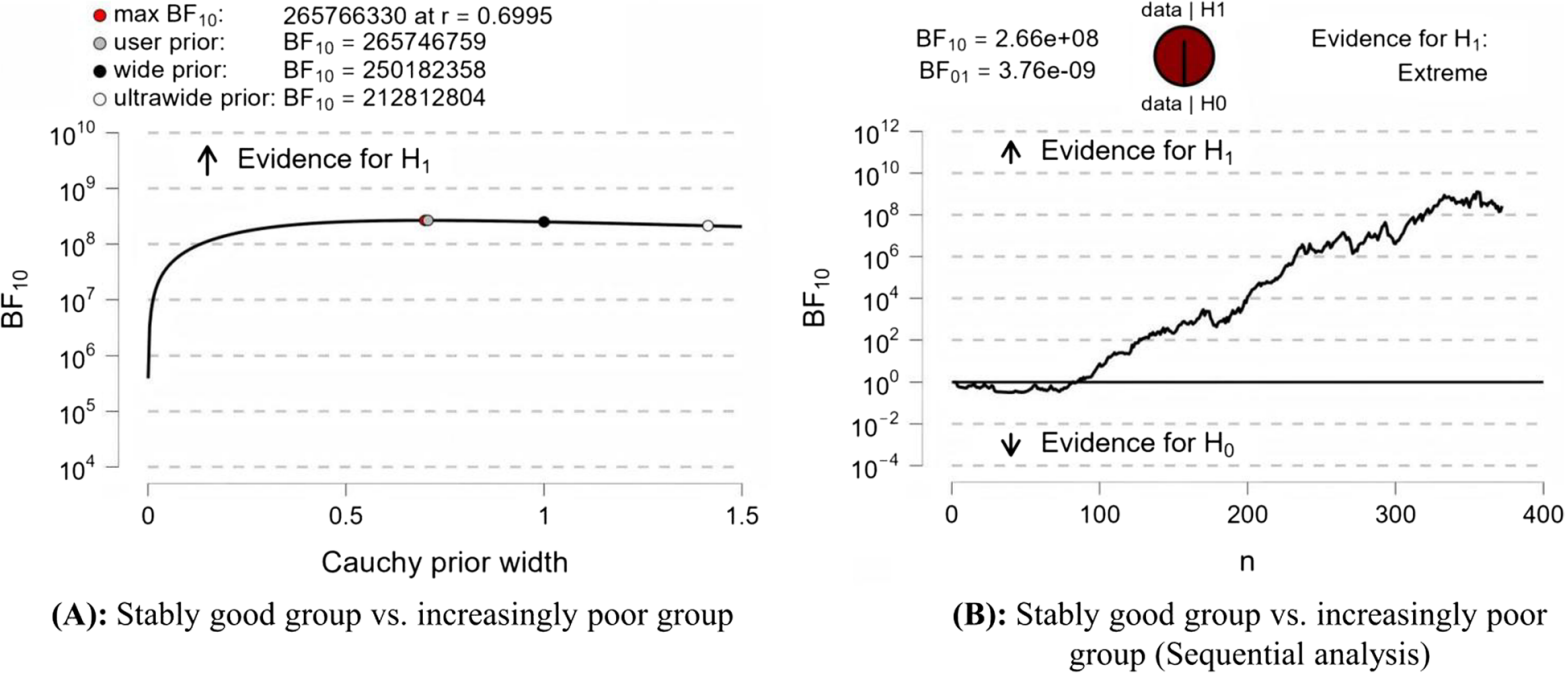
Bayesian independent sample *t*-test analysis

A notable difference in EPDS scores was found between the stably good and increasingly poor groups (BF10 = 2.66e + 08, Fig. 3). As shown in Table 4, the postpartum depression rate was significantly elevated in the increasingly poor trajectory compared to the stably good trajectory (OR = 2.60, 95% CI [1.58, 4.28], *P* < 0.001). This link remained significant after adjusting for age, level of education, work status, birthplace, marital status, monthly household income, nulliparity, pregnancy intention, and mode of delivery (adjusted OR = 2.75, 95% CI [1.62, 4.66], *P* < 0.001). Results of the linear regression (Table 5) show that the crude and adjusted coefficients did not differ statistically, whether the total EPDS score included item 7 or not.

**Table 4:** Associations of sleep quality trajectories with postpartum depression

	Crude OR (95% CI)	*P*	Adjusted OR^a^ (95% CI)	*P*
Stably good	1 (reference)		1 (reference)	
Increasingly worse	2.60 (1.58, 4.28)	< 0.001	2.75 (1.62, 4.66)	< 0.001

*Abbreviations*: *CI* confidence interval, *OR* odds ratio. ^a^Age, level of education, work status, birthplace, marital status, monthly household income, nulliparity, pregnancy intention, and mode of delivery were adjusted

**Table 5:** Comparison of regression coefficients with and without inclusion of EPDS item 7

	Crude model	Adjusted model^a^
B (all items)	2.99***	3.05***
B (without item 7)	2.50***	2.54***
SE (all items)	0.44	0.45
SE (without item 7)	0.39	0.40
*z*-value for difference^b^	0.83	0.86
P for difference	0.40	0.39

*B* unstandardized coefficients, *SE* standard error. ***P < 0.001. ^a^Adjusted model was adjusted for age, education, employment status, monthly household income, place of birth, nulliparity, pregnancy intent, and delivery mode. The reference group was set as stably good trajectory. ^b^ ${\mathrm{z}}=\frac{{\textrm{B1}}-{\textrm{B2}}}{\sqrt{{\textrm{SE1}}^{2} +{\textrm{SE2}}^{2}}}$, where B represents the regression coefficients and SE represents the standard errors

### Network characteristics

**Fig. 4:**
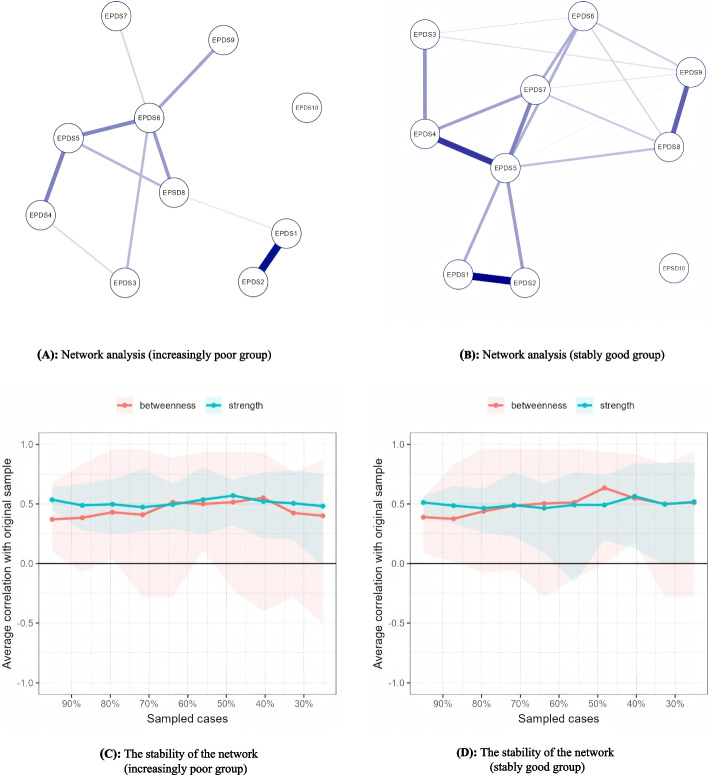
Ising network analysis. **A** Network analysis (increasingly poor group). **B** Network analysis (stably good group). **C** The stability of the network (increasingly poor group). **D** The stability of the network (stably good group)

The network visualizations are shown in Fig. 4. In the increasingly poor trajectory (Fig. 4A), the core symptom was EPDS6 (strength = 1.839), while in the stably good trajectory (Fig. 4B), EPDS5 (strength = 2.055) was identified as the core symptom. The stability test reveals that the centrality stability coefficient is close to 0.5, indicating that the network stability is relatively good (see Fig. 4C & D). Comparison between the stably good and increasingly poor groups revealed statistically significant differences (*P* = 0.039, see Additional file 1: Fig. S1).

### Computer-simulated intervention

**Fig. 5:**
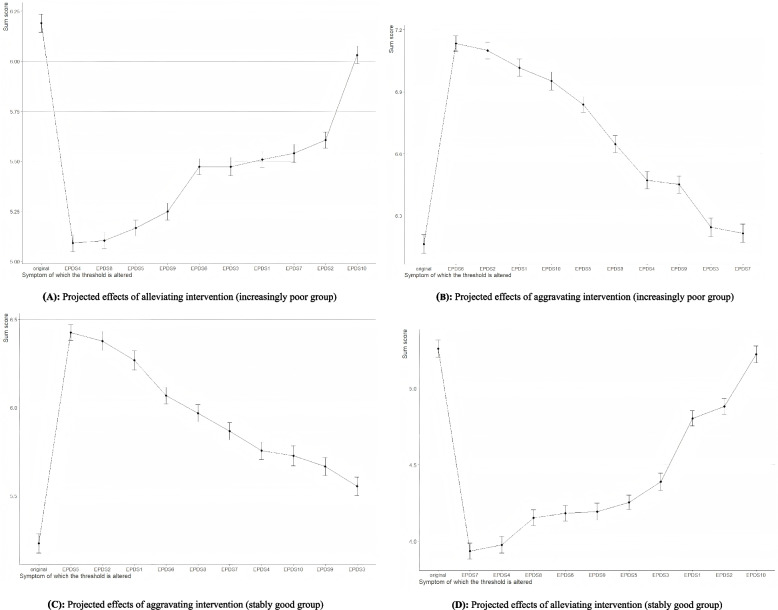
The effect of computer-simulated interventions on postpartum depressive symptoms

As depicted in Fig. 5, the impact of individual symptoms on network dynamics varied between the two trajectories. In the increasingly poor group, EPDS4 was associated with a decline in the predictive symptom total score from 6.15 to 5.10 (Fig. 5A). Conversely, EPDS6, identified as the core symptom, exhibited the strongest predictive effect, raising the sum score from 6.15 to 7.13 (Fig. 5B). In the stably good group, EPDS5, identified as the core symptom, produced the greatest predictive increase in the sum score from 6.15 to 7.13 (Fig. 5C). Then, EPDS7 contributed to a decrease in the predicted sum score, lowering it from 5.26 to 3.94 (Fig. 5D).

## Discussion

This study examined the longitudinal trajectories of sleep quality across pregnancy and the early postpartum period, examining their association with PPD. Two distinct trajectories were identified, each exhibiting unique pathways in the manifestation of postpartum depressive symptoms. These findings may guide the creation of targeted interventions tailored to the specific needs.

Consistent with prior studies [8], the identification of two distinct sleep quality trajectories suggests heterogeneous sleep patterns during pregnancy and the postpartum period. However, unlike previous research that identified four sleep patterns, namely, stably poor, mild increasingly poor, high increasingly poor, and stably good [10], our study revealed only “stably good” and “increasingly poor” groups. Notably, the proportion of women in the “stably good” group (58.6%) was higher than that reported in the studies by Sedov et al. and Tomfohr et al. [9, 10]. This discrepancy may be due to variations in sample characteristics, measurement intervals, or the instruments used for assessment. Both prior studies were conducted in developed countries. While Sedov et al. employed measurement intervals similar to ours, they used the Insomnia Severity Index with a substantially smaller sample size of 142 participants [9]. In contrast, Tomfohr et al. used the same PSQI but assessed participants at two prenatal points, followed by at 3 and 6 months postpartum [10].

Our findings reinforce the established link between declining sleep quality and an elevated risk of PPD [40]. Specifically, women with worsening sleep quality are over twice as likely to develop PPD, highlighting the urgent need for ongoing monitoring of sleep quality and the development of targeted interventions to maintain healthy sleep patterns during this critical period.

We also gained novel insights into the symptom structure of PPD across different trajectories. The “increasingly poor” group exhibited distinct core symptoms (EPDS6) characterized by feelings of overwhelm and an inability to cope. This pattern is consistent with previous findings that chronic poor sleep quality is related to reduced coping capacity to manage daily demands, leading to a pervasive feeling of being overwhelmed [41, 42]. Additionally, poor sleep quality is commonly acknowledged as a risk factor for heightened stress perception, which may exacerbate the effects of other perinatal stressors [43]. Conversely, the “stably good” group displayed prominent panic or acute fear. This may suggest that even in the absence of chronic sleep problems, some women experience heightened anxiety or panic symptoms during the postpartum period. Such symptoms could reflect individual differences in stress reactivity or preexisting vulnerabilities to anxiety, which may be triggered or intensified by perinatal physiological stressors [44]. In this context, panic becomes the primary expression of PPD, distinguishing it from the cognitive overwhelm seen in the “increasingly poor” group.

Furthermore, the differential effectiveness of interventions targeting specific symptoms underscores the context-dependent characteristics of PPD networks. The prominent alleviation associated with EPDS4 (“generalized anxiety”) in the “increasingly poor” group underscores its central role in this phenotype. Chronic poor sleep quality may impair the ability to disengage attention from negative stimuli, potentially transforming “unfounded” anxiety into a central hub of distress symptoms [45]. Additionally, ongoing anxiety and stress have also been linked to greater difficulties in mood regulation, which may exacerbate depressive symptoms [46]. In contrast, the alleviating effect of EPDS7 (mood-related sleep difficulties) in the “stably good” group highlights the reciprocal relationship between sleep and depression [47]. In this context, sleep issues may be more situational and directly linked to negative emotions, rather than being chronic. Acute sleep disturbances triggered by emotional distress may initiate a reflex-like physiological stress response, leading to exaggerated emotional reactivity and heightened negative mood in the moment [48]. This creates a feedback loop where unhappiness and sleep issues reinforce each other, ultimately increasing the overall depressive burden [49].

### Strengths and limitations

The longitudinal design and implementation of network analysis represent key strengths of this research. Nevertheless, certain limitations warrant consideration. First, the study was conducted in a single geographic region, and the sample predominantly consisted of middle-class participants, which may limit generalizability. Second, our study relied on the PSQI to measure sleep quality, which lacks more objective indicators and may reduce the accuracy of sleep monitoring. Third, the binary categorization may not adequately reflect the intensity of symptoms, highlighting the necessity for more advanced models to encompass a wider spectrum of symptoms. Lastly, we did not include other variables that may affect sleep quality and PPD, such as the levels of social support and depression during pregnancy.

### Implications

The findings have important implications. Future research should integrate objective sleep measurements, like wearable technology, in conjunction with self-report instruments to minimize bias and enhance accuracy [50]. It is also crucial to account for factors like prenatal complications, social support levels, and depression to fully understand their effects on PPD. Clinically, routine monitoring of sleep quality could help identify women at risk for depression, enabling early intervention. Tailored interventions based on sleep trajectory subgroups may enhance treatment efficacy. For the “stably good” group, strategies should prioritize emotional regulation and expression, as evidence from a randomized controlled trial indicates that affect regulation training can significantly reduce depressive symptoms [51]. Conversely, for the “increasingly poor” group, integrating sleep management and problem-solving is crucial, as cognitive behavioral therapy for insomnia has proven effective in alleviating both insomnia and depression [52].

## Conclusions

In summary, we identified distinct trajectories of sleep quality during pregnancy and the postpartum period and revealed unique pathways of postpartum depressive symptoms between the “stably good” and “increasingly poor” groups. These findings may empower clinicians to devise more effective, symptom-specific strategies customized to meet the specific needs of postpartum women.

## Supplementary Information

Additional file 1: Fig. S1. Result of network comparison test.

## Data Availability

The data that support the findings of this study are available on request from the corresponding author. The data are not publicly available due to privacy or ethical restrictions.

## Abbreviations

ANOVA: Analysis of variance
AIC: Akaike information criterion
LL: Log-likelihood
BIC: Bayesian information criterion
EPDS: Edinburgh Postnatal Depression Scale
OR: Odds ratios
PPD: Postpartum depression
PSQI: Pittsburgh Sleep Quality Index
SD: Standard deviations
