# Supplementary material for: Association between trajectories of sleep quality and postpartum depression: a group-based trajectory model and computer-simulated network analysis
Source: BMC Med. 2026 Feb 10;24:161. doi: 10.1186/s12916-026-04689-z (PMC12990398; doi:10.1186/s12916-026-04689-z)
Supplement: Supplementary file 1 — Additional file 1: Fig. S1. Result of network comparison test. [file 12916_2026_4689_MOESM1_ESM.docx]

**Additional file 1: Figure S1**

**Fig. S1.** Result of network comparison test

| 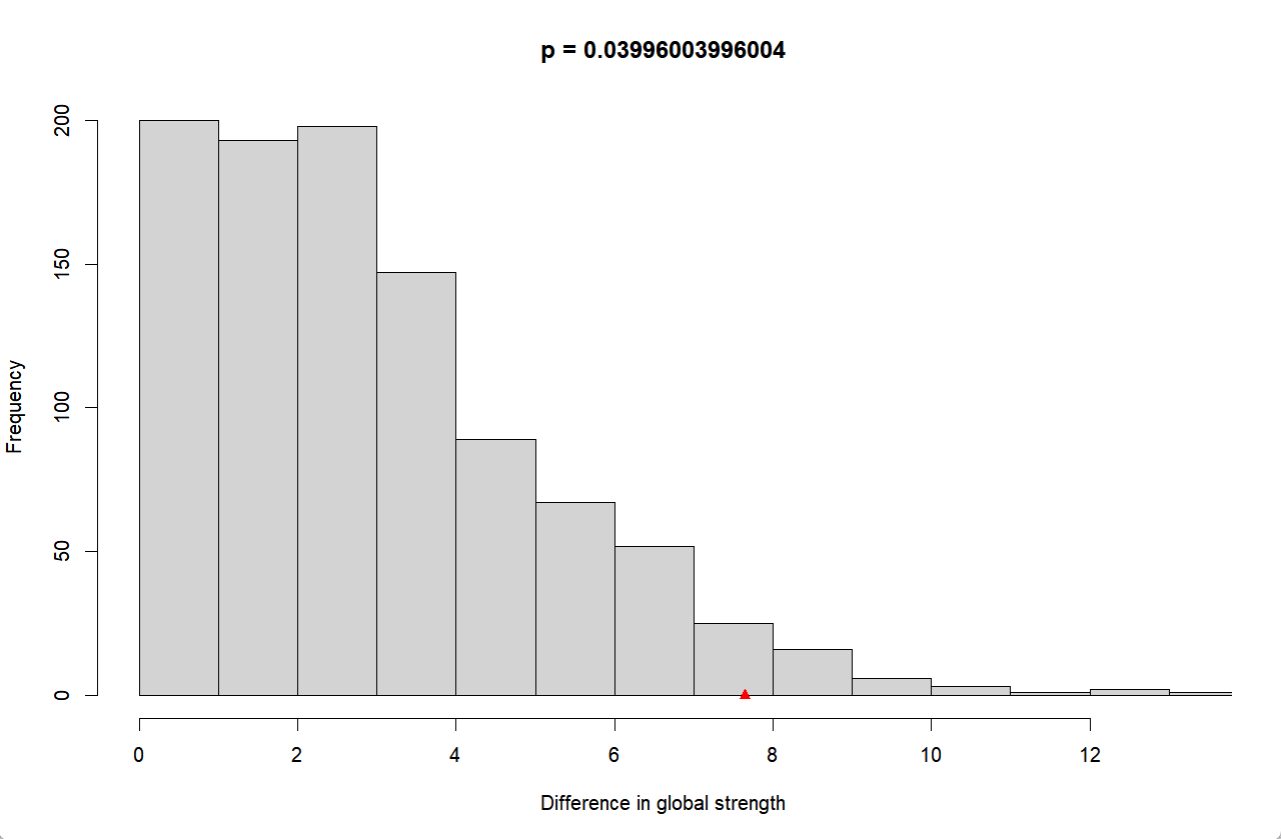 |
| --- |
| **Figure S1.** Result of network comparison test |
